# EFFECTS OF AN IMMERSIVE VIRTUAL REALITY TAI CHI INTERVENTION ON THE MENTAL HEALTH OF OLDER ADULTS

**DOI:** 10.1093/geroni/igae098.3961

**Published:** 2024-12-31

**Authors:** Jaehyun Kim, Marcos Ardon Lobos, Serim Park, Richard Kim, Jinsil Seo, Junhyoung Kim

**Affiliations:** East Carolina University, Greenville, North Carolina, United States; East Carolina University, Greenville, North Carolina, United States; Texas A&M University, College Station, Texas, United States; St. Mark’s School, Southborough, Massachusetts, United States; Texas A&M University, College Station, Texas, United States; Texas A&M University, College Station, Texas, United States

## Abstract

A significant proportion of the older population (approximately 1 in 5) suffers from diagnosed and sub-clinical mental health conditions. However, evidence indicates that older adults are less likely to seek mental health services than younger adults, primarily due to stigma and limited mental health literacy. As researchers have highlighted the urgent need for innovative solutions in geriatric mental health care, we explored the effectiveness of the use of an immersive virtual reality Tai Chi intervention (IVTC) as a potential solution. Eighteen older adults from senior centers in North Carolina, United States participated in eight 15-minute IVTC sessions over a month and completed surveys at the start (T1) and end (T2) of the program. Analysis using two-tailed paired t-tests revealed significant reductions in depressive symptoms following participation in the IVTC (t(17) = 4.594; p <.001; SE =.12), with a large effect size (d = 1.10). Additionally, perceived stress levels were significantly decreased after participation (t(17) = 2.541; p <.001; SE =.09), showing a moderate effect size (d =.60). Participants also reported high usability scores, indicating a favorable response to use of the IVTC program. These findings suggest that the IVTC program effectively reduces the stress and depressive symptoms of older adults, highlighting its potential as an innovative, technology-based intervention for those at higher risk of developing mental disorders. Study findings also provide important implications for future research and emphasize the need for further exploration of IVTC as a promising approach to geriatric mental health care.

